# Brain Structural Networks Associated with Intelligence and Visuomotor Ability

**DOI:** 10.1038/s41598-017-02304-z

**Published:** 2017-05-19

**Authors:** Youngwoo Bryan Yoon, Won-Gyo Shin, Tae Young Lee, Ji-Won Hur, Kang Ik K. Cho, William Seunghyun Sohn, Seung-Goo Kim, Kwang-Hyuk Lee, Jun Soo Kwon

**Affiliations:** 1 Department of Brain and Cognitive Sciences, Seoul National University, Seoul, 08826 Republic of Korea; 2 Medical Research Center, Seoul National University Hospital, Seoul, 03080 Republic of Korea; 3 Department of Psychology, Chung-Ang University, Seoul, 06974 Republic of Korea; 4 Max Planck Institute for Human Cognitive and Brain Sciences, Leipzig, 04103 Germany; 5 Department of Psychiatry, Seoul National University College of Medicine, Seoul, 03080 Republic of Korea

## Abstract

Increasing evidence indicates that multiple structures in the brain are associated with intelligence and cognitive function at the network level. The association between the grey matter (GM) structural network and intelligence and cognition is not well understood. We applied a multivariate approach to identify the pattern of GM and link the structural network to intelligence and cognitive functions. Structural magnetic resonance imaging was acquired from 92 healthy individuals. Source-based morphometry analysis was applied to the imaging data to extract GM structural covariance. We assessed the intelligence, verbal fluency, processing speed, and executive functioning of the participants and further investigated the correlations of the GM structural networks with intelligence and cognitive functions. Six GM structural networks were identified. The cerebello-parietal component and the frontal component were significantly associated with intelligence. The parietal and frontal regions were each distinctively associated with intelligence by maintaining structural networks with the cerebellum and the temporal region, respectively. The cerebellar component was associated with visuomotor ability. Our results support the parieto-frontal integration theory of intelligence by demonstrating how each core region for intelligence works in concert with other regions. In addition, we revealed how the cerebellum is associated with intelligence and cognitive functions.

## Introduction

The biological underpinning of intelligence and cognitive ability has long been of interest to neuroscientists^1^. Early approaches, such as brain lesion studies, focused on specific regions of the brain and linked those regions to intelligence and cognitive function^2–4^. The development of neuroimaging techniques enabled neuroscientists to investigate the human brain *in vivo*, and evidence obtained from neuroimaging studies suggests that no single brain region has an exclusively dominant effect on intelligence^5, 6^. However, an extensive literature review supported the parieto-frontal integration theory, which claims that the parietal cortex and the frontal cortex are the core regions involved in intelligence^7^. The parieto-frontal integration theory has been substantiated by various neuroimaging studies, including focal brain lesion^8, 9^ and magnetic resonance spectroscopy studies^10^. Among structural neuroimaging studies, more than 40% have indicated that both frontal and parietal regions are correlated with intelligence^7^. Recent neuroimaging studies have investigated the neural basis of intelligence at the network level. Studies using diffusion tensor imaging (DTI) have revealed that the structural organization of white matter (WM) at the network level^11^ and its developmental trajectory^12^ are important in intelligence. Resting-state functional magnetic resonance imaging (MRI) studies have indicated that interactions between parietal and frontal regions are important for intellectual functioning^13–15^. However, to date, the network-level underpinnings of intelligence have been derived primarily from WM structural or functional MRI studies.

There is firm evidence that grey matter (GM) is associated with intelligence^6, 16, 17^. However, there is a limited number of studies demonstrating how the GM structural network is linked to intelligence. Previous studies have used the graph theoretical approach to assess how GM regions form structural networks^18–21^. However, for the graph theoretical analysis, the region of interest must be determined prior data analysis. Thus, other important regions that could potentially contribute to intelligence, such as the cerebellum, are often overlooked^18^. To extract structural networks without any prior assumption, we applied a recently developed approach, source-based morphometry (SBM), to structural MRI to examine the relationship of GM structural networks to intelligence and cognitive functions. SBM applies independent component analysis (ICA) to a segmented image and arranges voxels into sets that contain similar information^22^. Whereas the voxel-based morphometry (VBM) approach is a univariate approach that automatically segments brain images into voxelwise measures of GM^23^, SBM, the multivariate extension of VBM, acquires common morphological features of GM concentration among individuals at the network level and thus provides us with structural networks. We expect that SBM will enable us to uncover submerged regional networks within the brain and further provide us with a more comprehensive picture of GM structural networks. Another advantage of SBM is that this method can remove sources arising from artefacts. There is some disagreement among studies that have used VBM to demonstrate an association between intelligence and brain structure. While Gong *et al*. reported a correlation between intelligence and medial prefrontal GM concentration^17^, Colom *et al*. found that intelligence was associated with GM concentration in diverse brain regions, more significantly for temporal and occipital areas than for the frontal lobes^24^. We suggest that the brain networks subserving intelligence may be better identified with a less noisy source of interest, which can be acquired from SBM.

In addition to investigating the relationship between structural networks and intelligence, we investigated structural networks in relation to cognitive functions. Verbal fluency, processing speed, and executive functioning have been reported to be particularly relevant to brain structural networks^25–30^. However, this relevance has not been shown at GM structural network level. To assess the cognitive functions, we administered the Trail Making Test (TMT) and the Controlled Oral Word Association Test (COWAT) to the participants. The TMT has been reported to measure cognitive functions and consists of two parts: Part A (TMT-A), which is known to evaluate visual search and processing speed^31^, and Part B (TMT-B), which is known to assess set-shifting and controlled attention ability^32^. The COWAT, one of the most frequently applied tests used to assess verbal fluency^33^, is also known to measure verbal working memory^34^. The cognitive functions were further compared with the GM structural network.

We hypothesized that intelligence is associated with parietal and frontal GM structural network and that different structural networks interact with different domains of cognitive functions. To test this hypothesis, we first acquired GM structural networks using SBM. After acquiring structural networks, we investigated the relationship between the structural networks and intelligence. In addition, we explored how cognitive functions, assessed with the TMT and COWAT, are associated with the GM structural network.

## Results

### Demographic and Cognitive Characteristics

Ninety-two healthy participants aged 17 to 48 years, with intelligence quotients (IQs) ranging from 83 to 137, were included in the study. The descriptive statistics of the demographic and cognitive characteristics are presented in Table 1. There were no gender differences in demographics (e.g., age, IQ, and education year) and in neuropsychological data, except for the number of errors made in both parts A and B of TMT. Statistics on gender differences in demographic and cognitive characteristics are summarized in Supplementary Table S1.

**Table 1:** The statistics for the demographic and cognitive characteristics of the study sample. IQ: Intelligence quotient, TMT: Trail Making Test, M: Male, F: Female, COWAT: Controlled Oral Word Association Test. ^a^Estimated IQ was measured by the short form of the K-WAIS.

		Mean	SD
Age (years)		26.10	6.87
Gender (M/F)		54/38	
Education (years)		14.61	1.77
Estimated IQ^a^		113.90	11.79
TMT	Part A (seconds)	23.06	6.97
(M: 50/F: 33)	Part B (seconds)	54.83	17.59
	Part B - Part A (seconds)	31.77	15.67
	Part A (# of errors)	0.14	0.39
	Part B (# of errors)	0.27	0.52
COWAT	Category (No. of responses)	42.32	8.91
(M: 42/F: 29)	Letter (No. of responses)	45.15	10.09

### Component Estimation and Comparison with Functional Networks

**Figure 1:**
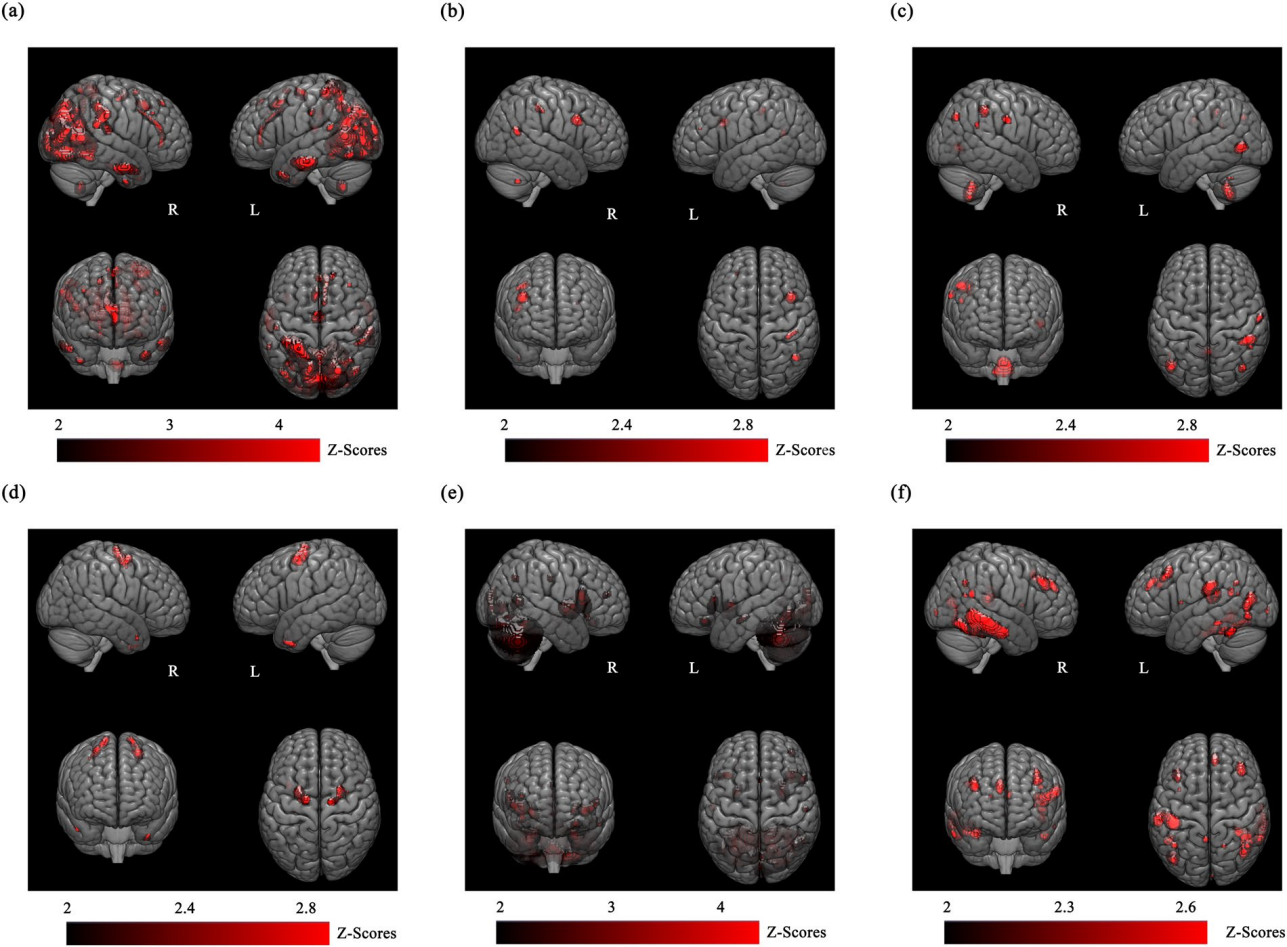
The structural components discovered by source-based morphometry. (**a**) Precuneus component; (**b**) fronto-temporal component; (**c**) cerebello-parietal component; (**d**) frontal component; (**e**) cerebellar component; (**f**) temporal component. All displayed networks had a threshold of Z > 2. The colour bar indicates the Z-score (of the contribution of each voxel to the component).

Six components were extracted from the GM images according to the minimum description length criteria (Fig. 1 and Supplementary Table S2)^35^. There were no significant gender differences in structural components (see Supplementary Table S1). All the components were visually inspected, and 5 out of 6 components agreed with the functional MRI-based networks. As the scale (including the sign) of the components cannot be determined by the ICA, we used an absolute value of a correlation coefficient for the comparison. Similar to the findings by Smith *et al*.^36^, the maximal absolute correlation coefficients between the functional MRI networks and SBM components in the present study were as follows: |R| = 0.55 for the “visual” network (Map 1_20_) and precuneus component; |R| = 0.28 for the “auditory” network (Map 7_20_) and fronto-temporal component; |R| = 0.33 for the “executive control” network (Map 8_20_) and cerebello-parietal component; |R| = 0.34 for the “default mode network” (Map 4_20_) and frontal component; |R| = 0.34 for the “cerebellum” network (Map 5_20_) and cerebellar component; and |R| = 0.10 for the “sensorimotor” network (Map 6_20_) and temporal component (see Supplementary Fig. S1). Although the Bonferroni-corrected P-values were extremely small due to the large number of voxels, one SBM component showed negligible agreement with the resting state network (RSN) (|R| = 0.10). Five other SBM components showed high agreement (between 0.28 and 0.55) with the known RSNs. This result is consistent with the previous SBM studies that have reported high correspondence between the structural and functional covariance structures^37, 38^.

### Relationship between Structural Networks and Neuropsychological Data

**Figure 2:**
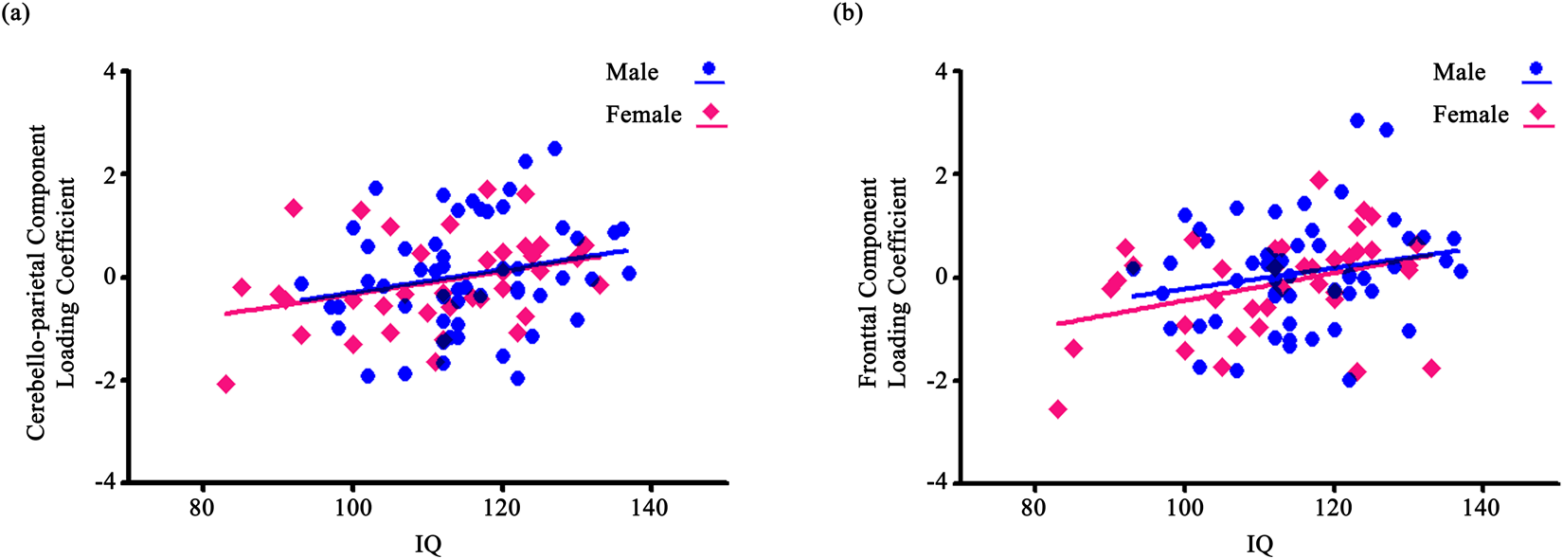
The association between structural networks and intelligence quotient (IQ). (**a**) Correlation between intelligence and the cerebello-parietal component; (**b**) correlation between intelligence and the frontal component.

**Figure 3:**
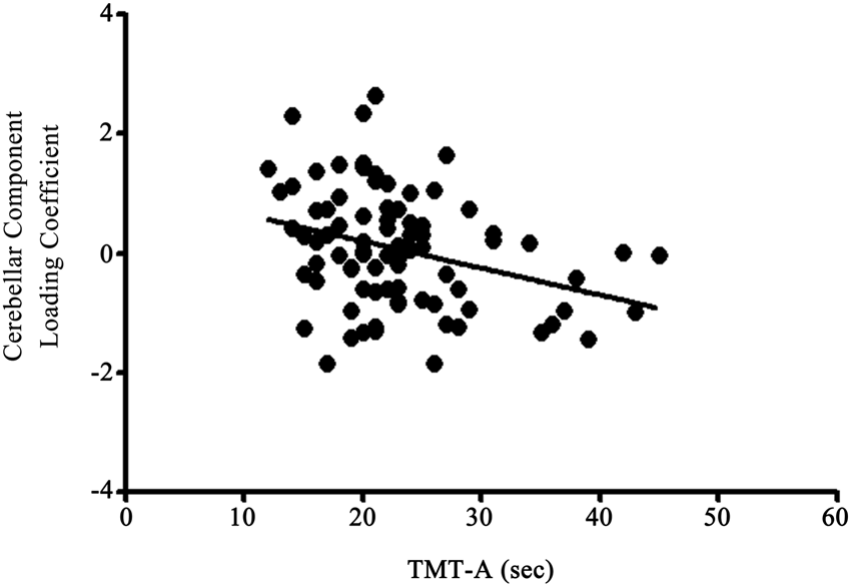
The association between the cerebellar component and results from the Trail Making Test, Part A (TMT-A).

The correlation analysis revealed that individuals’ intelligence was associated with the cerebello-parietal component (R = 0.264, P = 0.011) (see Supplementary Fig. S1c) and the frontal component (R = 0.288, P = 0.005) (see Supplementary Fig. S1d). When the associations were separately analysed for males and females, females demonstrated a significant positive association in both the cerebello-parietal component (R = 0.330, P = 0.043) and the frontal component (R = 0.371, P = 0.022), whereas males did not show such associations (the cerebello-parietal component: R = 0.217, P = 0.116; the frontal component: R = 0.207, P = 0.134) (Fig. 2). The cerebello-parietal component and the frontal component were significantly correlated (R = 0.727, P < 0.001). When we controlled for the influence of the cerebello-parietal component on intelligence, there was no significant association between the frontal component and intelligence (R = 0.141, P = 0.182). The same was observed when the influence of the frontal component was considered for the correlation analysis between the cerebello-parietal component and intelligence (R = 0.075, P = 0.483). Multiple regression analysis predicted loading coefficients of the cerebellar component (F_(1, 81)_ = 9.314, P = 0.003), with an R² = 0.321. The completion time (in seconds) of TMT-A (Beta = −0.321, P = 0.003) significantly contributed to the multiple regression model (Fig. 3). The completion time (in seconds) of neither TMT-B nor B-A significantly contributed to multiple regression models predicting loading coefficients of any structural component. In addition, we observed a marginally significant association between the cerebello-parietal component and the number of responses from the letter fluency of the COWAT (R = 0.235, P = 0.052).

## Discussion

Our study examined GM structural networks by applying a multivariate morphometric procedure and further revealed the networks’ relationships with intelligence and visuomotor ability (TMT-A). Our results showed that the cerebello-parietal component and the frontal component were associated with intelligence. To our knowledge, this is the first study to link GM anatomical connectivity between the parietal cortex and cerebellum in humans *in vivo* and to further associate this structural connectivity with intelligence. In addition, the cerebellar component was associated with TMT-A, which measures visual search ability and processing speed. By determining the morphometric variation patterns of the brain through the use of SBM, we demonstrated how the core regions are associated with other brain regions and contribute to intelligence.

Five of the 6 structural networks were spatially similar to previously reported functional networks. In multimodal studies in which ICA was applied across different modalities, the structural networks were comparable to the networks extracted from DTI and functional MRI studies^38, 39^. Similarly, in our structural components, we observed characteristics that were assumed to be unique to the functional networks. Our cerebello-parietal component and the frontal component demonstrated patterns similar to the executive control network and the default-mode network, respectively. Both functional networks, the executive control network and the default-mode network, were reported to be associated with intelligence^14, 40^. Functional MRI indirectly measures neuronal cell activity, which mainly occurs in GM^41^. However, only a few studies have established the association between the GM structural networks and functional networks^38, 42, 43^. By linking the GM structural networks to the functional networks and further to intelligence and cognitive functions, our results shed light on the importance of GM structural investigation at the network level.

Cerebellum and parietal regions form the cerebello-parietal component, which was associated with intelligence. In primates, an anatomical connection between the parietal and pontine regions was revealed^44^, and the pons was further shown to be extensively connected with cerebellar cortical areas via the middle cerebellar peduncle^45^. This WM parieto-ponto-cerebellar tract was also revealed in human studies using DTI^46^. During phonological storage processing, co-activation of the cerebellar and parietal regions was reported^47^. In addition, during verbal encoding, transcranial direct current stimulation over the cerebellum affected the interaction between the cerebellum and the parietal cortex^48^. Both studies emphasize the importance of the cerebellar-parietal connection on the processes supporting verbal intelligence. In accordance with previous reports linking the cerebello-parietal network and verbal intelligence, we observed a marginally significant association between the cerebello-parietal component and verbal fluency. In addition, it is interesting to note that our structural analysis is consistent with other functional studies with regards to the more detailed anatomical characteristics of the cerebellum. In a resting-state functional MRI study, Buckner *et al*. investigated associations between the cerebellar and cerebral regions^49^. The study revealed that the default-mode network, lateral temporal cortex, and inferior parietal lobule were functionally connected with Crus I and II in the cerebellar cortex. Previous studies indicated the potential influence of this network on higher-level cognition^49–51^, and our cerebello-parietal component, composed of Crus II and the inferior parietal lobule, was associated with both intelligence and verbal fluency. Although many studies have reported that frontal and parietal regions play a critical role in intelligence, some studies have emphasized the importance of the cerebellum^52–55^. As noted by Jung and Haier^7^, relatively overlooked brain regions, such as the cerebellum, are also important for brain function, and the importance of the cerebellum in intelligence was revealed in our results.

The frontal component partially includes middle and superior temporal gyri of temporal region. For decades, many studies have reported that the frontal lobe is related to intelligence^56–58^, and to a lesser degree, it is also reported that the temporal lobe is involved in intelligence^59^. In the functional MRI and DTI studies that assessed brain regional network aspects, it is reported that the connectivity between frontal and temporal is related to cognitive function^60–62^, and that linkage abnormality between the two regions may be related to the pathophysiology of the mental illness^63, 64^. A previous study reported the influence of the language network, composed of frontal and temporal regions, on not only semantic but also syntactic processing^65^. We suggest that the intricate interaction between semantic and syntactic processing affects intelligence, and the interaction was reflected in the frontal component at GM structural level in our study.

Both the cerebello-parietal component and the frontal component are associated with intelligence. However, when the cerebello-parietal component was controlled for, the association between the frontal component and intelligence became non-significant. The same was observed in the relationship between the cerebello-parietal component and intelligence when the frontal component was controlled for. Hence, we suggest that two networks are intimately related in contributing to processes involved in intelligence. The parieto-frontal integration theory proposed two important brain regions associated with intelligence, and our results further suggest that both regions work in concert with different regions in the brain to support intelligence. It is interesting to note that the negative weighting of the cerebello-parietal component included the frontal regions, while the negative weighting of the frontal component included the parietal regions (see Supplementary Table S2). The negative weighting region of the SBM component means that the region has negative contributions to the structural network, indicating that the region demonstrates an opposite trend of GM concentration to the positively weighted regions^39^. By demonstrating an antithetical pattern of parietal and frontal interaction in both intelligence-related structural networks, we speculate that there may be a more complex mechanism underlying intelligence that cannot be monolithically explained by increased or decreased GM regional density. We suggest that greater GM density does not always indicate a higher intelligence level. How effectively neural cells are organized^66, 67^ and how efficiently brain regions interact each other may influence intelligence, but this possibility must be further explored.

We found that TMT-A performance was associated with the cerebellar component. The cerebellar component comprised of mainly cerebellar sensorimotor areas (lobules IV, V, and VI) along with occipital visual areas. Hence, it is not surprising to find an association between the cerebellar component and performance on the TMT-A that measures visual search ability and processing speed^31^. Strong evidence has recently emerged of a much more fundamental role for the cerebellum in higher cognitive processing than was previously considered^53, 68^. Accordingly, cerebellar volume changes were observed in patients with various mental disorders^69–71^, and that those with cerebellar damage had cognitive abnormalities in various domains^72, 73^. In this light, one might expect the cerebellar component to be associated with TMT-B (or TMT-B minus TMT-A), which measures the ability to control attention and set-shifting^32^. However, we did not find such an association. Thus, our results highlight the specificity of the cerebellar component for sensorimotor function measured with the TMT-A.

There are some limitations of our study. First, although some existing studies have applied SBM to explore structural networks by estimating structural covariance^74, 75^, more studies are needed to further validate this relatively new approach. Because our results were consistent with previous studies regarding the relationship between brain regions and cognition, the present study provides additional face validity for the SBM approach. Second, neuropsychological assessment tests were not applied to all participants. The performance of SBM analysis is expected to be better with more data^22^. For instance, the number of components that could be extracted is proportional to the number of participants^35^. Therefore, we tried to include more participants in our study, but missing data due to administration issues hindered the inclusion of more participants (e.g., the TMT outcome variables for 9 out of 92 participants and the COWAT outcome variables for 21 out of 92 participants were not available). We anticipate that a large amount of data will help us to extract more biologically interpretable sources that are beneficial in revealing structural networks related to the complex mechanisms of intelligence and cognition.

In summary, our multivariate morphometric analysis revealed structural networks consistent with previously reported functional networks. Among the extracted structural networks, the cerebello-parietal component and the frontal component were associated with intelligence. The multivariate approach enabled us to emphasize the role of a previously overlooked region, the cerebellum, in intelligence in conjunction with the parietal lobe. To our knowledge, this study is the first to link GM structural connectivity between parietal and cerebellum regions and to further link this connectivity with intelligence. In addition, the cerebellum was involved in verbal fluency within the cerebello-parietal component and visuomotor processing speed in conjunction with visual areas. By emphasizing the role of the frontal and parietal lobes in intelligence, our results provide additional information on how the cerebellum is associated with intelligence and visuomotor ability at the structural network level. Based on our study’s findings, future studies should investigate the association of the brain’s structural networks with intelligence and cognitive function using various approaches.

## Methods

### Participants

Ninety-two participants were selected from the subject pool of prospective cohort study projects at the Seoul National University Hospital and the Department of Brain and Cognitive Sciences at Seoul National University. The participants were recruited from the community via internet advertisement and screened with the Structured Clinical Interview of the DSM-IV, Non-Patient Version (SCID-NP)^76^. To control for any possible confounding variables and to only include mentally healthy individuals in the present study, exclusion criteria consisted of the following: (i) head injury, medical or neurological disorders, or any substance abuse; (ii) IQ below 80; (iii) less than 17 years of age; (iv) less than 10 years of education; or (v) any first- or second-degree relatives with a history of any psychiatric illness. During recruitment, only participants with the appropriate quality of MRI data were included in our cohort after the MRI data quality check, which was conducted by 2 independent researchers. Specifically, participants with MRI signal loss due to orthodontic appliances (n = 2) or dental implants (n = 1), were not included in our current dataset. Written informed consent was obtained from all participants after they had been completely informed of the study protocols. This study was approved by the Institutional Review Board of Seoul National University Hospital and all methods were performed in accordance with the relevant guidelines and regulations.

### Neuropsychological Data

Intelligence was assessed with the short form of the Korean version of the Wechsler Adult Intelligence Scale (WAIS), which consists of Vocabulary, Arithmetic, Block Design, and Picture Arrangement subtests^77^. In addition, the TMT was administered to 83 participants. The TMT is composed of two parts: (i) TMT-A involves sequentially connecting numbers with a continuous line; (ii) TMT-B involves alternately connecting numbers and letters. The total number of errors and the response time (in seconds) were recorded. The COWAT^78^ was administered to 71 participants to measure their ability to generate words within a specific category and to name words beginning with a certain letter. The number of responses during the time limit (within 1 minute) was recorded. TMT (n = 9) or COWAT (n = 21) data were not available for 21 participants, as they were from our different neuroimaging studies. Nevertheless, these individuals were included because they followed identical MRI and IQ assessment procedures as with the remainder of participants.

### Image Acquisition

Structural images were obtained with a Siemens 3 T Trio MRI scanner (Siemens Magnetom Trio, Erlangen, Germany) using a 12-channel head coil. The T1-weighted anatomical image was acquired using magnetization-prepared rapid gradient echo imaging (echo time [TE]/repetition time [TR] = 1.89/1670 ms, field of view [FOV] = 250 mm, flip angle = 9°, matrix = 256 × 256, voxel size = 1.0 × 1.0 × 1.0 mm^3^, 208 slices). The time required to collect the structural scan was 3 minutes and 54 seconds.

### Image Preprocessing

Structural images were preprocessed according to the VBM pipeline^79^ in the Computational Anatomy Toolbox (CAT; http://dbm.neuro.uni-jena.de/cat/), which runs in the Statistical Parametric Mapping toolbox version 12 (SPM12; http://www.fil.ion.ucl.ac.uk/spm/). The toolbox segments a native structural image into GM, WM, and cerebrospinal fluid (CSF), based on the maximum a posteriori approach, which does not require information about the tissue probability prior to segmentation^80^. Then, affine registration to MNI space was applied, and the non-linear deformation parameter was subsequently calculated using the high-dimensional Dartel algorithm^81^. To remove noise, a multithreaded denoising filter was applied^82^. As suggested in previous studies^22, 83^, the GM images were smoothed with a full width at half maximum (FWHM) Gaussian kernel of 12 mm.

### SBM Analysis

SBM applies ICA to segmented and smoothed structural images from the preprocessing step^22^. ICA was calculated using the Infomax algorithm implemented in the GIFT Toolbox (http://mialab.mrn.org/software/gift/). The number of estimated components was determined according to the minimum description length criteria^35^. We used ICASSO^84^ and repeated the estimation 20 times with bootstrapping to increase the stability of the estimated components. Each of the preprocessed GM volumes was separately converted into a one-dimensional vector. Ninety-two preprocessed images were arrayed into a GM image matrix in the dimension of the number of subjects by the number of GM voxels. Then, the image matrix was decomposed into a mixing matrix and a source matrix. As it was estimated according to the minimum description length criteria^35^, six components were extracted. Thus, the 92 subject-by-the number of GM voxels matrix was decomposed to a 92 subject-by-6 components matrix (mixing matrix) and a 6 component-by-GM voxels matrix (source matrix). The mixing matrix involves loading coefficients that demonstrate how each structural component contributes to the 92 subjects and thus contains information about the relationship between each subject and each component. The (Z-transformed) loading coefficients were further used to investigate the association between structural components and neuropsychological variables. The source matrix demonstrates how each component contributes to different GM voxels and thus involves spatial information about the structural components. The (Z-transformed) source matrix was used for visualization and labelling of structural components. Geographical information of each component identified in the source matrix was used in the visual inspection step to exclude artefact components in further analyses.

### Comparison with Functional Networks

It has been demonstrated that the GM-based components are spatially well matched to the resting-state fMRI-based components^35, 36^. To validate that the source separation in our data was also biologically meaningful, each component extracted from SBM was compared with previously reported functional networks^36^. The functional networks were composed of 10 well-matched maps that were independently derived from both the activation meta-analysis and resting-state data^36^. The component maps were downloaded from (http://fsl.fmrib.ox.ac.uk/analysis/brainmap+rsns/), and a detailed description of how the functional connectivity maps were obtained can be found in Smith *et al*.^36^. The components extracted from the SBM and functional network components were compared based on spatial cross-correlation (i.e., Pearson’s correlation between vectorized images).

### Statistical Analysis

The associations between the GM structural components and neuropsychological data were estimated. As the measure of intelligence was already adjusted for the effect of age and gender, Pearson’s correlation analysis was applied to find the association between intelligence and structural connectivity loading coefficient values. In addition, for the structural networks that were significantly associated with intelligence, we conducted the Pearson’s correlation analysis separately for males and females. To control for the effect of age and gender, partial correlation analysis was applied to find the association between the COWAT outcome values and the loading coefficient values of each structural component. Because the TMT-A reflects visual processing abilities and the TMT-B reflects working memory and set-shifting abilities, the response time of TMT-B was subtracted by TMT-A (B-A) to remove the effect of processing speed ability and to observe executive control abilities^85^. Multiple regression analyses were conducted to examine the relationship between structural networks and TMT outcomes, including completion time (in seconds) of TMT and number of errors made during TMT. Each multiple regression analysis was performed with the loading coefficient of each structural component as the dependent variable. Independent variables consisted of TMT outcomes, including completion time of TMT and number of errors made during TMT, as well as other factors that could affect the TMT outcome, such as age and gender. Each of the TMT outcomes, TMT-A, TMT-B, and B-A, was analysed separately using different multiple regression analyses. All statistical analyses were performed using IBM SPSS Statistics for Windows, version 22.0 (IBM Corp., Armonk, NY).

## Electronic supplementary material

Supplementary Information
