# Supplementary material for: Brain Structural Networks Associated with Intelligence and Visuomotor Ability
Source: Sci Rep. 2017 May 19;7:2177. doi: 10.1038/s41598-017-02304-z (PMC5438383; doi:10.1038/s41598-017-02304-z)
Supplement: Supplementary file 1 — Supplementary Information [file 41598_2017_2304_MOESM1_ESM.pdf]

**Brain Structural Networks Associated with Intelligence and Visuomotor Ability**

## (Supplementary Information)

Youngwoo Bryan Yoon[1], Won-Gyo Shin[1], Tae Young Lee[2], Ji-Won Hur[3], Kang Ik K. Cho[2], William Seunghyun Sohn[2], Seung-Goo Kim[4], Kwang-Hyuk Lee[2], Jun Soo Kwon[1,2,5,*]

[1] Department of Brain and Cognitive Sciences, Seoul National University, Seoul, 08826, Republic of Korea

[2] Medical Research Center, Seoul National University Hospital, Seoul, 03080, Republic of Korea

[3] Department of Psychology, Chung-Ang University, Seoul, 06974, Republic of Korea

[4] Max Planck Institute for Human Cognitive and Brain Sciences, Leipzig, 04103, Germany

[5] Department of Psychiatry, Seoul National University College of Medicine, Seoul, 03080, Republic of Korea

## Corresponding Author Contact Information

Professor Jun Soo Kwon

E-mail: kwonjs@snu.ac.kr

Tel +82-2-2072-2972

Fax +82-2-747-9063

**Supplementary Table S1. Gender differences in demographics, cognitive characteristics, and structural components.** IQ: Intelligence quotient, TMT: Trail Making Test, M: Male, F: Female, COWAT: Controlled Oral Word Association Test. [a]Estimated IQ was measured using the short form of the K-WAIS.

| | | Male | Female | *t* | *P* |
|---|---|---|---|---|---|
| *Demographics* | | | | | |
| Age (years) | | 26.04 ± 6.22 | 26.18 ± 7.78 | -0.101 | 0.920 |
| Education (years) | | 14.61 ± 1.69 | 14.61 ± 1.90 | 0.016 | 0.988 |
| *Cognitive Characteristics* | | | | | |
| Estimated IQ[a] | | 115.28 ± 10.49 | 111.95 ± 13.32 | 1.340 | 0.184 |
| TMT | Part A (sec) | 23.38 ± 7.19 | 22.58 ± 6.71 | 0.512 | 0.610 |
| (M: 50 / F: 33) | Part B (sec) | 57.04 ± 18.41 | 51.48 ± 15.96 | 1.417 | 0.160 |
| | Part B - Part A (sec) | 33.66 ± 16.26 | 28.91 ± 14.51 | 1.359 | 0.178 |
| | Part A (# of errors) | 0.22 ± 0.46 | 0.03 ± 0.17 | 2.621 | 0.011 |
| | Part B (# of errors) | 0.38 ± 0.57 | 0.09 ± 0.38 | 2.767 | 0.007 |
| COWAT | Category (No. of responses) | 43.29 ± 9.43 | 40.93 ± 8.06 | 1.096 | 0.277 |
| (M: 42 / F: 29) | Letter (No. of responses) | 45.14 ± 9.74 | 45.17 ± 10.74 | -0.012 | 0.990 |
| *Structural Components* | | | | | |
| Precuneus Component | | -0.03 ± 1.12 | 0.04 ± 0.81 | -0.316 | 0.753 |
| Fronto-temporal Component | | 0.05 ± 1.03 | -0.07 ± 0.97 | 0.525 | 0.601 |
| Cerebello-parietal Component | | 0.05 ± 1.07 | -0.07 ± 0.89 | 0.535 | 0.594 |
| Frontal Component | | 0.09 ± 1.03 | -0.13 ± 0.96 | 1.011 | 0.315 |
| Cerebellar Component | | 0.02 ± 1.07 | -0.03 ± 0.91 | 0.242 | 0.809 |
| Temporal Component | | 0.11 ± 1.09 | -0.16 ± 0.85 | 1.280 | 0.204 |

**Supplementary Figure S1. The structural components (in red) and the corresponding functional networks from Smith *et al*. (in green).** Overlapping regions are depicted in yellow. (**a**) Precuneus component; (**b**) fronto-temporal component; (**c**) cerebello-parietal component; (**d**) frontal component; (**e**) cerebellar component; (**f**) temporal component. Both displayed networks had a threshold of |Z| > 2. Each component's association with intelligence quotient (IQ) is presented in the scatter diagram. Axial slices are presented at z = -63, -54, -44, -35, -26, -16, -7, 2, 12, 22, 30, 40, 50, 58, 68, and 78.

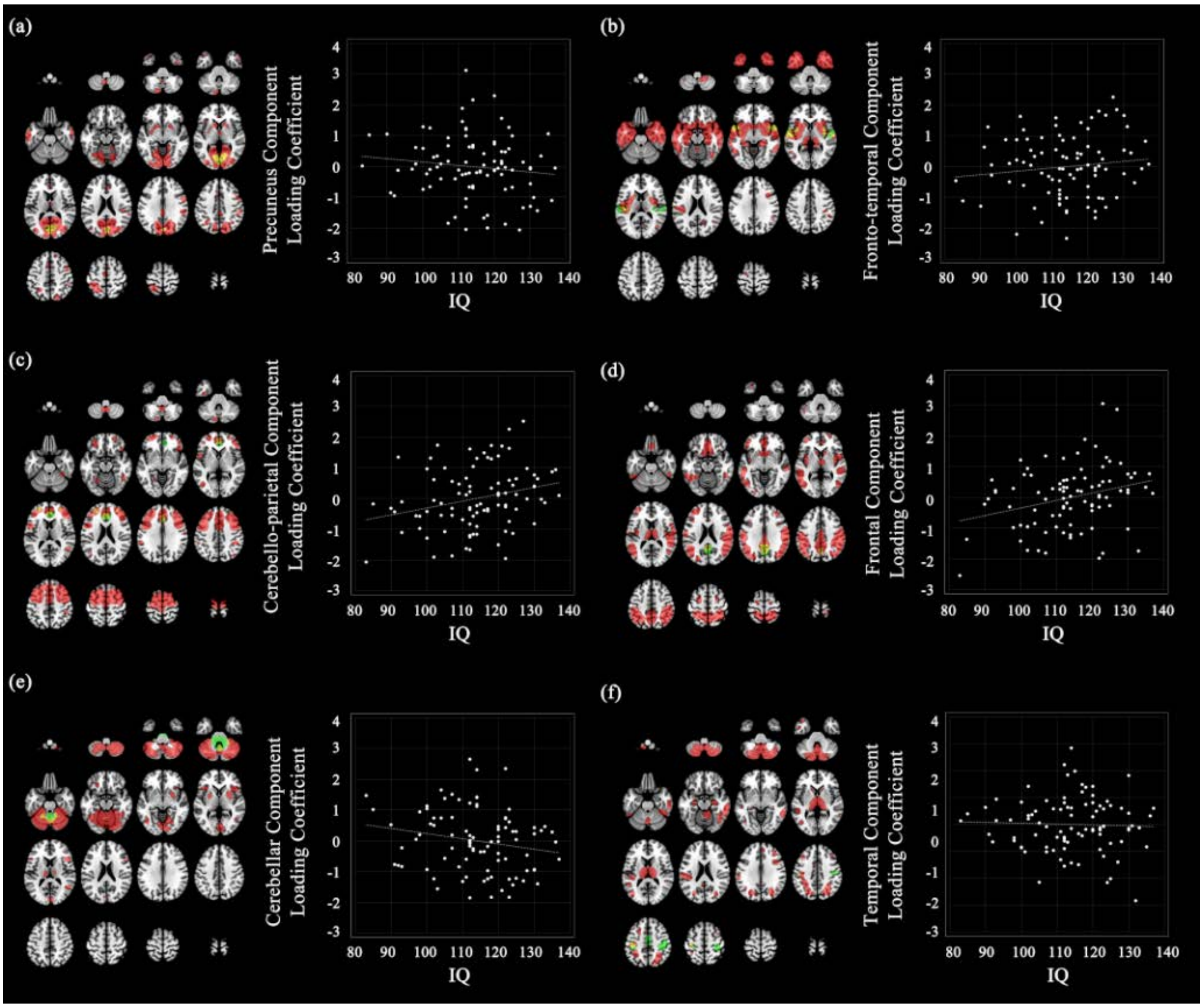

**Supplementary Table S2. Anatomical description of the SBM components.** Voxels above the threshold of Z > 2.5 within each component are presented. Note that only regions with positive contributions to the loading weights are summarized. Anatomical descriptions were acquired from the Talairach Daemon (http://www.talairach.org/daemon.html). For the cerebellar regions, the agreed-upon names were labelled according to Schmahmann[57]. na: no strong (Z > 2.5) contribution of the component.

| Anatomical regions | Brodmann Area | Volume (in cc) for left / right hemispheres. | Random effects: Max Value (x, y, z) for left / right hemispheres. |
|---|---|---|---|
| *Precuneus Component* | | | |
| Posterior Cingulate | 23, 29, 30, 31 | 4.6 / 5.8 | 6.7 (-16, -59, 11) / 7.2 (21, -56, 10) |
| Cuneus/visual cortex | 7, 17, 18, 23 | 8.0 / 10.6 | 6.3 (-4, -74, 13) / 6.3 (1, -76, 20) |
| Precuneus | 7, 19, 23, 31 | 5.0 / 8.1 | 6.0 (-13, -63, 29) / 5.2 (15, -62, 34) |
| Lingual Gyrus | 17, 18, 19 | 8.1 / 4.4 | 4.9 (-4, -77, 5) / 4.6 (18, -54, 4) |
| Parahippocampal Gyrus | 30 | 0.2 / 0.8 | 3.7 (-19, -51, 4) / 4.7 (21, -51, 4) |
| Middle Occipital Gyrus | 18, 19 | 0.6 / 1.5 | 2.9 (-31, -86, 3) / 4.0 (36, -77, 11) |
| Inferior Temporal Gyrus | 20, 21 | 0.9 / 0.0 | 3.7 (-58, -16, -21) / na |
| Postcentral Gyrus | 2, 5, 40 | 1.3 / 0.0 | 3.7 (-30, -42, 59) / na |
| Inferior Parietal Lobule | 40 | 0.7 / 0.3 | 3.5 (-34, -38, 57) / 2.8 (55, -29, 32) |
| Superior Parietal Lobule | 5, 7 | 0.8 / 0.0 | 3.4 (-27, -45, 59) / na |
| Culmen (IV/V) | | 0.3 / 0.0 | 3.3 (-12, -66, -6) / na |
| Middle Temporal Gyrus (Posterior) | 39 | 0.0 / 0.6 | na / 3.2 (40, -75, 11) |
| Fusiform Gyrus | 19, 20 | 0.4 / 0.0 | 3.2 (-55, -16, -23) / na |
| Declive (VI) | | 0.1 / 0.0 | 2.7 (-18, -66, -11) / na |
| Inferior Semi-Lunar Lobule (Crus II) | | 0.1 / 0.0 | 2.6 (-3, -57, -40) / na |
| *Fronto-temporal Component* | | | |

| | | | |
|---|---|---|---|
| Middle Frontal Gyrus | 9 | 0.0 / 0.5 | na / 4.0 (37, 14, 27) |
| Superior Temporal Gyrus | 22 | 0.0 / 0.4 | na / 3.4 (43, -54, 19) |
| Postcentral Gyrus | 2 | 0.0 / 0.3 | na / 3.0 (37, -29, 41) |
| Middle Temporal Gyrus (Posterior) | 39 | 0.0 / 0.1 | na / 2.8 (40, -54, 22) |
| Cerebellar Tonsil (IX) | | 0.0 / 0.3 | na / 2.7 (43, -54, -33) |
| | | | |
| *Cerebello-parietal Component* | | | |
| Cerebellar Tonsil (IX) | | 0.8 / 0.6 | 3.9 (0, -56, -38) / 3.7 (3, -54, -40) |
| Inferior Parietal Lobule | 40 | 0.0 / 1.7 | na / 3.8 (45, -36, 42) |
| Inferior Semi-Lunar Lobule (Crus II) | | 0.3 / 0.2 | 3.6 (-3, -59, -39) / 3.6 (3, -60, -41) |
| Middle Occipital Gyrus | 19, 37 | 0.7 / 0.0 | 3.5 (-43, -67, 5) / na |
| Inferior Temporal Gyrus (Posterior) | 37 | 0.4 / 0.0 | 3.2 (-43, -70, 2) / na |
| Precuneus | 19, 39 | 0.0 / 0.6 | na / 3.1 (40, -68, 37) |
| Middle Temporal Gyrus (Posterior) | 37 | 0.3 / 0.0 | 3.0 (-43, -70, 8) / na |
| Precentral Gyrus | 4 | 0.0  0.3 | na / 2.9 (59, -12, 31) |
| Tuber (VIIAt) | | 0.1 / 0.0 | 2.6 (-45, -57, -27) / na |
| | | | |
| *Frontal Component* | | | |
| Middle Frontal Gyrus | 6 | 1.5 / 1.0 | 3.2 (-25, 0, 50) / 2.9 (18, -10, 61) |
| Medial Frontal Gyrus | 6 | 0.2 / 0.1 | 2.9 (-19, 3, 52) / 2.7 (10, -10, 65) |
| Middle Temporal Gyrus (Anterior) | 21, 38 | 0.5 / 0.2 | 2.8 (-34, 3, -35) / 2.6 (49, 9, -26) |
| Superior Temporal Gyrus (Anterior) | 38 | 0.1 / 0.1 | 2.6 (-39, 13, -32) / 2.7 (46, 10, -30) |
| | | | |
| *Cerebellar Component* | | | |
| Tuber (VIIAt) | | 3.0 / 4.5 | 5.9 (-43, -57, -26) / 8.7 (43, -55, -28) |
| Culmen (IV/V) | | 9.0 / 9.9 | 6.7 (-31, -55, -20) / 8.5 (40, -55, -25) |
| Cerebellar Tonsil (IX) | | 2.1 / 5.9 | 4.8 (-42, -53, -31) / 8.0 (43, -57, -31) |

| | | | |
|---|---|---|---|
| Declive (VI) | | 9.0 / 9.9 | 6.5 (-31, -59, -20) / 7.8 (37, -56, -22) |
| Uvula (IX) | | 1.7 / 2.2 | 5.5 (-31, -62, -23) / 6.5 (33, -62, -23) |
| Pyramis (VIII) | | 1.9 / 2.1 | 4.0 (-36, -70, -31) / 5.8 (43, -66, -32) |
| Inferior Semi-Lunar Lobule (Crus II) | | 1.0 / 3.3 | 3.4 (-42, -63, -35) / 5.3 (40, -62, -36) |
| Fusiform Gyrus | 19, 20, 37 | 1.3 / 1.5 | 4.2 (-40, -55, -17) / 5.1 (39, -53, -18) |
| Lingual Gyrus | 17, 18 | 0.8 / 1.3 | 3.1 (-22, -69, -10) / 3.0 (19, -82, -12) |
| Lentiform Nucleus (Putamen) | | 0.0 / 0.4 | na / 3.0 (25, 4, 1) |
| Middle Occipital Gyrus | 18 | 0.0 / 0.1 | na / 2.9 (27, -82, -10) |
| Inferior Frontal Gyrus | 45, 47 | 0.0 / 0.1 | na / 2.9 (42, 21, 4) |
| Insula | 13 | 0.0 / 0.3 | na / 2.8 (37, 22, 3) |
| Cuneus | 17, 18 | 0.0 / 0.2 | na / 2.8 (3, -87, 13) |
| Middle Temporal Gyrus (Posterior) | 39 | 0.0 / 0.1 | na / 2.7 (49, -55, 12) |
| Cerebellar Lingual (I/II) | | 0.0 / 0.1 | na / 2.7 (9, -47, -13) |
| | | | |
| *Temporal Component* | | | |
| Middle Temporal Gyrus | 20, 21, 37, 39 | 0.7 / 4.6 | 3.5 (-42, -71, 12) / 4.0 (59, -36, -11) |
| Superior Temporal Gyrus | 41 | 0.6 / 0.0 | 3.8 (-40, -31, 17) / na |
| Inferior Parietal Lobule | 40 | 1.0 / 0.0 | 3.6 (-58, -25, 25) / na |
| Middle Occipital Gyrus | 19 | 0.7 / 0.0 | 3.6 (-42, -68, 9) / na |
| Inferior Temporal Gyrus | 20, 21, 37 | 0.0 / 1.6 | na / 3.5 (59, -30, -16) |
| Middle Frontal Gyrus | 8, 9 | 0.3 / 0.8 | 2.9 (-36, 22, 38) / 3.5 (36, 33, 27) |
| Transverse Temporal Gyrus | 41 | 0.3 / 0.0 | 3.5 (-40, -30, 13) / na |
| Postcentral Gyrus | 40 | 0.7 / 0.0 | 3.4 (-52, -22, 19) / na |
| Insula | 13 | 0.9 / 0.0 | 3.3 (-40, -28, 19) / na |
| Fusiform Gyrus | 20, 37 | 0.0 / 1.5 | na / 3.1 (34, -40, -18) |
| Medial Frontal Gyrus | 9 | 0.0 / 0.4 | na / 3.0 (6, 44, 25) |
| Culmen (IV/V) | | 0.1 / 0.2 | 2.7 (-46, -49, -22) / 2.8 (34, -37, -21) |

| | | | |
|---|---|---|---|
| Declive (VI) | | 0.1 / 0.0 | 2.8 (-48, -52, -19) / na |
| Posterior Cingulate | 23 | 0.1 / 0.0 | 2.5 (-3, -48, 22) / na |

**Supplementary Table S3. Anatomical description of the cerebello-parietal and frontal components.** Voxels above the threshold of |Z| > 2.5 within each component are presented. Note that only regions with negative contributions to the loading weights are summarized. Anatomical descriptions were acquired from the Talairach Daemon (http://www.talairach.org/daemon.html). na: no strong (|Z| > 2.5) contribution of the component.

| Anatomical regions | Brodmann Area | Volume (in cc) for left / right hemispheres. | Random effects: Max Value (x, y, z) for left / right hemispheres. |
|---|---|---|---|
| *Cerebello-parietal Component* | | | |
| Middle Frontal Cortex | 8, 9, 10, 46 | 14.3 / 23.5 | 5.8 (-40, 8, 36) / 6.3 (39, 4, 47) |
| Inferior Frontal Gyrus | 6, 9, 44, 46 | 3.1 / 2.8 | 5.4 (-43, 7, 33) / 5.3 (45, 9, 31) |
| Precentral Gyrus | 6, 44 | 3.4 / 2.9 | 5.4 (-43, 5, 36) / 5.4 (40, 6, 37) |
| Superior Frontal Gyrus | 6, 8, 9, 10 | 5.6 / 6.6 | 4.3 (-15, -4, 67) / 4.7 (25, 9, 53) |
| Medial Frontal Gyrus | 8, 9, 10, 32 | 3.1 / 3.8 | 3.8 (-13, -4, 62) / 4.1 (6, 34, 31) |
| Anterior Cingulate | 24, 32 | 0.5 / 1.0 | 3.1 (0, 28, 32) / 4.0 (4, 36, 29) |
| *Frontal Component* | | | |
| Cingulate Gyrus | 23, 24, 31 | 5.2 / 4.7 | 5.9 (0, -42, 37) / 6.2 (3, -39, 36) |
| Precuneus | 7, 19, 31, 39 | 6.0 / 6.0 | 5.6 (-3, -48, 34) / 5.5 (3, -48, 34) |
| Inferior Parietal Lobule | 39, 40 | 2.1 / 6.9 | 3.5 (-42, -30, 40) / 4.7 (39, -39, 45) |
| Paracentral Lobule | 4, 5, 7, 31 | 1.2 / 1.3 | 4.2 (-1, -41, 49) / 4.1 (6, -44, 56) |
| Precentral Gyrus | 4, 6 | 0.2 / 2.9 | 3.2 (-53, -17, 35) / 4.0 (59, -7, 28) |
| Postcentral Gyrus | 2, 3, 5, 40, 43 | 2.7 / 7.0 | 3.8 (-49, -24, 37) / 4.0 (43, -27, 43) |
| Superior Temporal Gyrus | 13, 22, 39 | 2.3 / 2.8 | 3.9 (-43, -56, 26) / 3.7 (50, -36, 7) |
| Middle Temporal Gyrus | 19, 21, 37 | 3.6 / 1.8 | 3.5 (-43, -59, 24) / 3.8 (45, -57, 22) |
| Supramarginal Gyrus | 40 | 1.4 / 0.6 | 3.7 (-45, -55, 30) / 3.5 (48, -50, 22) |
| Superior Parietal Lobule | 5, 7 | 0.0 / 1.0 | na / 3.6 (33, -46, 51) |
| Angular Gyrus | 39 | 0.6 / 0.4 | 3.4 (-45, -58, 33) / 3.4 (42, -67, 31) |

| | | | |
|---|---|---|---|
| Thalamus | | 0.2 / 0.8 | 3.1 (-1, -10, 7) / 3.4 (3, -10, 7) |
| Inferior Temporal Gyrus | 37 | 0.5 / 0.0 | 3.2 (-48, -67, 2) / na |
| Middle Occipital Gyrus | 19 | 0.4 / 0.0 | 3.1 (-45, -70, 5) / na |
| Medial Frontal Gyrus | 11, 25 | 0.8 / 0.3 | 3.1 (-1, 25, -15) / 2.9 (1, 27, -13) |
| Insula | 13 | 0.0 / 0.3 | na / 2.8 (50, -24, 18) |
| Posterior Cingulate | 23, 31 | 0.1 / 0.0 | 2.6 (-3, -48, 23) / na |
